# 2-[Bis(5-chloro-2-pyridylamino)methyl]pyridine monohydrate

**DOI:** 10.1107/S1600536808035940

**Published:** 2008-11-22

**Authors:** Davar M. Boghaei, Mohammad Mahdi Najafpour, Vickie McKee

**Affiliations:** a Department of Chemistry, Sharif University of Technology, PO Box 11155-8639, Tehran, Iran; b Department of Chemistry, Loughborough University, Leicestershire LE11 3TU, England

## Abstract

In the title compound, the dihedral angles between the 2-amino-5-chloro­pyridyl rings and the pyridine ring are 56.26 (6)° and 78.83 (5)°; the angle between the 2-amino-5-chloro­pyridyl rings is 72.42 (5)°. The solvent water mol­ecules are linked to the organic compound by N—H⋯O, O—H⋯O, N—H⋯N and O—H⋯N hydrogen bonds. π⋯π Stacking inter­actions are also observed between the 2-amino-5-chloro­pyridyl rings (centroid⋯centroid distance = 3.243 Å).

## Related literature

For related crystallographic studies, see: Makowska-Grzyska *et al.* (2003 ▶); Li *et al.* (2008 ▶); Peori *et al.* (2008 ▶).
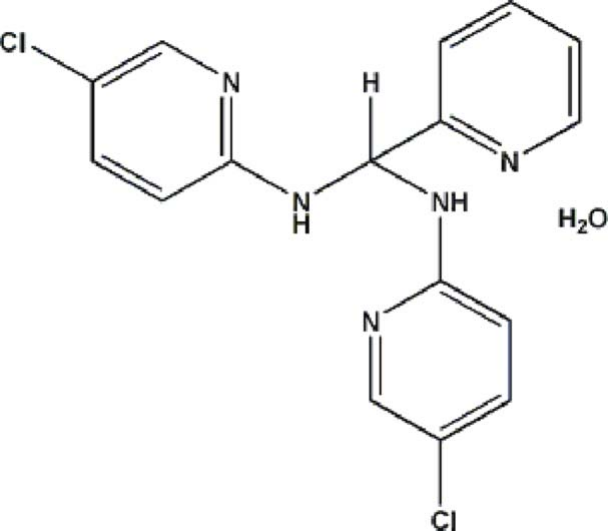


## Experimental

### 

#### Crystal data

C_16_H_13_Cl_2_N_5_·H_2_O
*M* *_r_* = 364.23
Monoclinic, 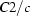

*a* = 34.414 (3) Å
*b* = 4.4337 (3) Å
*c* = 26.236 (2) Å
β = 124.778 (1)°
*V* = 3288.0 (4) Å^3^
*Z* = 8
Mo *K*α radiation
μ = 0.41 mm^−1^
*T* = 150 (2) K
0.35 × 0.10 × 0.05 mm

#### Data collection

Bruker APEXII CCD diffractometer
Absorption correction: multi-scan (*SADABS*; Sheldrick, 2003 ▶) *T*_min_ = 0.870, *T*_max_ = 0.980
14914 measured reflections
3778 independent reflections
2830 reflections with *I* > 2σ(*I*)
*R*_int_ = 0.042

#### Refinement

*R*[*F* ^2^ > 2σ(*F* ^2^)] = 0.039
*wR*(*F* ^2^) = 0.101
*S* = 1.04
3778 reflections
217 parameters
H-atom parameters constrained
Δρ_max_ = 0.34 e Å^−3^
Δρ_min_ = −0.25 e Å^−3^

### 

Data collection: *APEX2* (Bruker, 2005 ▶); cell refinement: *SAINT* (Bruker, 2005 ▶); data reduction: *SAINT*; program(s) used to solve structure: *SHELXS97* (Sheldrick, 2008 ▶); program(s) used to refine structure: *SHELXL97* (Sheldrick, 2008 ▶); molecular graphics: *SHELXTL* (Sheldrick, 2008 ▶); software used to prepare material for publication: *SHELXTL*.

## Supplementary Material

Crystal structure: contains datablocks I, global. DOI: 10.1107/S1600536808035940/wn2287sup1.cif

Structure factors: contains datablocks I. DOI: 10.1107/S1600536808035940/wn2287Isup2.hkl

Additional supplementary materials:  crystallographic information; 3D view; checkCIF report

## Figures and Tables

**Table 1:** Hydrogen-bond geometry (Å, °)

*D*—H⋯*A*	*D*—H	H⋯*A*	*D*⋯*A*	*D*—H⋯*A*
N2—H2*N*⋯N5^i^	0.87	2.15	2.994 (2)	165
N4—H4*N*⋯O1^ii^	0.87	2.18	2.993 (2)	156
O1—H1*O*⋯N1	0.82	1.98	2.773 (2)	164
O1—H2*O*⋯O1^iii^	0.82	1.96	2.758 (2)	163

Symmetry codes: (i) 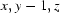; (ii) 
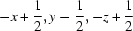
; (iii) 
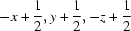
.

**Fig. 1.**
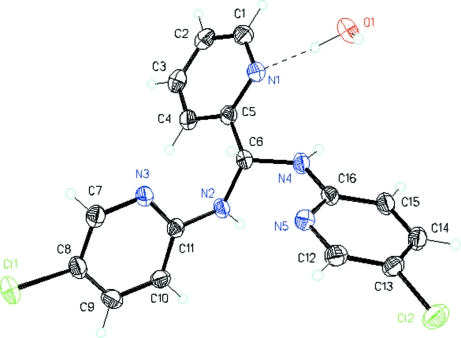
View of the molecular structure of the title compound, with displacement ellipsoids for non-H atoms drawn at the 50% probability level. The hydrogen atoms are represented by spheres of arbitrary radius. The hydrogen bond is shown as a dashed line.

**Fig. 2.**
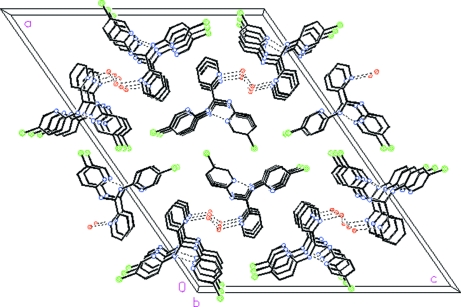
View of the crystal structure packing scheme. The dashed lines indicate hydrogen bonds. H atoms have been omitted.

**Fig. 3.**
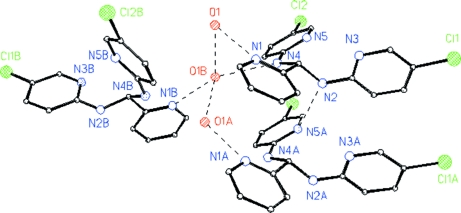
View of the hydrogen bonding (dashed lines) scheme. H atoms have been omitted.

## supplementary crystallographic information

### Comment

Primary amines add to imines to form aminal compounds; these are not stable (Peori *et al.*, 2008). The title compound, C_16_H_13_Cl_2_N_5_.H_2_O, was synthesized by the reaction of 2-amino-5-chloropyridine with 2-pyridinecarbaldehyde in ethanol. Tris(pyridyl)amines are very common ligands and many complexes containing such ligands have been reported (Makowska-Grzyska *et al.*, 2003; Li *et al.*, 2008).

In the title compound, intermolecular N—H···O and N—H···N hydrogen bonds involving amine NH and pyridyl groups form a two dimensional network (Table 1 and Fig. 3). π–π stacking interactions are also observed between the 2-amino-5-chloropyridyl rings [centroid···centroid distance = 3.243 Å]. The dihedral angles between the 2-amino-5-chloropyridyl rings and the pyridine ring are 56.26 (6)° and 78.83 (5)°; the angle between the  2-amino-5-chloropyridyl rings is 72.42 (5)°.

### Experimental

The title compound was synthesized by adding 2-pyridinecarbaldehyde (1 mmol, 107 mg) to a solution of 2-amino-5-chloropyridine (3 mmol, 386 mg) and manganese acetate (0.02 mmol, 4.90 mg) in ethanol (20 ml). The mixture was refluxed with stirring for 7 h. The resultant yellow solution was filtered. Colourless single crystals of the title compound suitable for *X*-ray structure determination were recrystallized from a mixture of ethanol / water (5/1) by slow evaporation of the solvents at room temperature over several days.

### Refinement

The H atoms bonded to C atoms were positioned geometrically and refined using the riding model with *U*_iso_(H) = 1.2*U*_eq_(parent atom); C—H = 0.95 and 1.00 Å for aromatic and methine C atoms, respectively. The H atoms bonded to N and O atoms were found in the difference Fourier map and refined with the following *DFIX* restraints: 0.87 (2) Å for N—H and 0.82 (2) Å for O—H. In the final refinement stages the AFIX3 constraint was used for these H atoms.

### Crystal data

**Table:**

C_16_H_13_Cl_2_N_5_·H_2_O	*F*_000_ = 1504
*M**_r_* = 364.23	*D*_x_ = 1.472 Mg m^−^^3^
Monoclinic, *C*2/*c*	Mo *K*α radiation λ = 0.71073 Å
Hall symbol: -C 2yc	Cell parameters from 2781 reflections
*a* = 34.414 (3) Å	θ = 2.4–26.1º
*b* = 4.4337 (3) Å	µ = 0.41 mm^−^^1^
*c* = 26.236 (2) Å	*T* = 150 (2) K
β = 124.778 (1)º	Lath, colourless
*V* = 3288.0 (4) Å^3^	0.35 × 0.10 × 0.05 mm
*Z* = 8	

### Data collection

**Table:**

Bruker APEXII CCD diffractometer	3778 independent reflections
Radiation source: fine-focus sealed tube	2830 reflections with *I* > 2σ(*I*)
Monochromator: graphite	*R*_int_ = 0.042
*T* = 150(2) K	θ_max_ = 27.5º
φ and ω scans	θ_min_ = 1.9º
Absorption correction: multi-scan(SADABS; Sheldrick, 2003)	*h* = −44→44
*T*_min_ = 0.870, *T*_max_ = 0.980	*k* = −5→5
14914 measured reflections	*l* = −34→34

### Refinement

**Table:**

Refinement on *F*^2^	Secondary atom site location: difference Fourier map
Least-squares matrix: full	Hydrogen site location: inferred from neighbouring sites
*R*[*F*^2^ > 2σ(*F*^2^)] = 0.039	H-atom parameters constrained
*wR*(*F*^2^) = 0.101	*w* = 1/[σ^2^(*F*_o_^2^) + (0.0476*P*)^2^ + 1.3212*P*] where *P* = (*F*_o_^2^ + 2*F*_c_^2^)/3
*S* = 1.04	(Δ/σ)_max_ < 0.001
3778 reflections	Δρ_max_ = 0.34 e Å^−^^3^
217 parameters	Δρ_min_ = −0.25 e Å^−^^3^
Primary atom site location: structure-invariant direct methods	Extinction correction: none

### Fractional atomic coordinates and isotropic or equivalent isotropic displacement parameters (Å^2^)

**Table:**

	*x*	*y*	*z*	*U*_iso_*/*U*_eq_	
N1	0.25729 (5)	0.5318 (4)	0.15040 (7)	0.0272 (3)	
C1	0.30054 (7)	0.4226 (5)	0.17347 (9)	0.0308 (4)	
H1	0.3259	0.4888	0.2134	0.037*	
C2	0.31028 (7)	0.2185 (4)	0.14231 (9)	0.0317 (4)	
H2	0.3414	0.1436	0.1606	0.038*	
C3	0.27344 (7)	0.1266 (5)	0.08383 (9)	0.0330 (4)	
H3	0.2789	−0.0104	0.0607	0.040*	
C4	0.22844 (7)	0.2366 (4)	0.05934 (9)	0.0279 (4)	
H4	0.2026	0.1759	0.0192	0.033*	
C5	0.22143 (6)	0.4366 (4)	0.09407 (8)	0.0232 (4)	
C6	0.17286 (6)	0.5693 (4)	0.06964 (8)	0.0235 (4)	
H6	0.1720	0.7800	0.0554	0.028*	
N2	0.13502 (5)	0.4001 (4)	0.01720 (6)	0.0244 (3)	
H2N	0.1224	0.2485	0.0239	0.029*	
N3	0.13945 (5)	0.6845 (3)	−0.05317 (7)	0.0254 (3)	
C7	0.12129 (7)	0.7555 (4)	−0.11245 (9)	0.0279 (4)	
H7	0.1368	0.9051	−0.1206	0.033*	
C8	0.08131 (6)	0.6224 (5)	−0.16202 (8)	0.0286 (4)	
Cl1	0.06183 (2)	0.72387 (14)	−0.23748 (2)	0.04370 (16)	
C9	0.05738 (6)	0.4096 (5)	−0.15136 (8)	0.0296 (4)	
H9	0.0295	0.3165	−0.1850	0.035*	
C10	0.07470 (6)	0.3361 (4)	−0.09131 (8)	0.0255 (4)	
H10	0.0588	0.1919	−0.0825	0.031*	
C11	0.11634 (6)	0.4768 (4)	−0.04267 (8)	0.0224 (4)	
N4	0.16586 (5)	0.5834 (4)	0.11881 (7)	0.0273 (4)	
H4N	0.1839	0.4738	0.1518	0.033*	
N5	0.10283 (5)	0.9191 (4)	0.06534 (7)	0.0267 (3)	
C12	0.06679 (6)	1.0659 (4)	0.06110 (9)	0.0288 (4)	
H12	0.0479	1.1975	0.0269	0.035*	
C13	0.05617 (6)	1.0341 (4)	0.10388 (9)	0.0275 (4)	
Cl2	0.008985 (17)	1.23175 (12)	0.09516 (3)	0.03644 (14)	
C14	0.08384 (7)	0.8454 (5)	0.15457 (9)	0.0298 (4)	
H14	0.0773	0.8215	0.1849	0.036*	
C15	0.12065 (7)	0.6949 (4)	0.16002 (9)	0.0281 (4)	
H15	0.1401	0.5650	0.1943	0.034*	
C16	0.12928 (6)	0.7355 (4)	0.11388 (8)	0.0238 (4)	
O1	0.27352 (5)	0.8741 (3)	0.24925 (6)	0.0359 (3)	
H1O	0.2644	0.7974	0.2157	0.043*	
H2O	0.2570	1.0234	0.2418	0.043*	

### Atomic displacement parameters (Å^2^)

**Table:**

	*U*^11^	*U*^22^	*U*^33^	*U*^12^	*U*^13^	*U*^23^
N1	0.0274 (8)	0.0316 (9)	0.0225 (8)	−0.0008 (7)	0.0142 (7)	0.0010 (7)
C1	0.0266 (10)	0.0366 (11)	0.0260 (10)	0.0014 (8)	0.0130 (8)	0.0040 (8)
C2	0.0286 (10)	0.0339 (11)	0.0381 (11)	0.0057 (8)	0.0224 (9)	0.0095 (9)
C3	0.0399 (11)	0.0315 (10)	0.0387 (11)	0.0032 (9)	0.0290 (10)	0.0017 (9)
C4	0.0304 (10)	0.0306 (10)	0.0253 (9)	−0.0008 (8)	0.0174 (8)	−0.0014 (8)
C5	0.0267 (9)	0.0239 (9)	0.0213 (8)	−0.0014 (7)	0.0150 (7)	0.0029 (7)
C6	0.0246 (9)	0.0281 (10)	0.0182 (8)	−0.0004 (7)	0.0125 (7)	−0.0008 (7)
N2	0.0275 (8)	0.0275 (8)	0.0187 (7)	−0.0047 (6)	0.0134 (6)	0.0003 (6)
N3	0.0272 (8)	0.0288 (8)	0.0216 (8)	−0.0025 (6)	0.0146 (7)	−0.0003 (6)
C7	0.0306 (10)	0.0302 (10)	0.0272 (9)	0.0009 (8)	0.0191 (8)	0.0033 (8)
C8	0.0303 (10)	0.0363 (11)	0.0195 (9)	0.0068 (8)	0.0144 (8)	0.0037 (8)
Cl1	0.0461 (3)	0.0610 (4)	0.0212 (2)	0.0011 (3)	0.0175 (2)	0.0074 (2)
C9	0.0245 (9)	0.0343 (11)	0.0235 (9)	0.0007 (8)	0.0099 (8)	−0.0018 (8)
C10	0.0210 (9)	0.0279 (10)	0.0268 (9)	−0.0003 (7)	0.0133 (8)	0.0004 (8)
C11	0.0235 (9)	0.0237 (9)	0.0222 (9)	0.0028 (7)	0.0144 (7)	−0.0010 (7)
N4	0.0276 (8)	0.0366 (9)	0.0191 (7)	0.0049 (7)	0.0142 (7)	0.0034 (7)
N5	0.0260 (8)	0.0317 (9)	0.0246 (8)	−0.0011 (7)	0.0157 (7)	0.0010 (7)
C12	0.0254 (9)	0.0319 (10)	0.0279 (10)	−0.0020 (8)	0.0145 (8)	0.0012 (8)
C13	0.0241 (9)	0.0293 (10)	0.0331 (10)	−0.0039 (8)	0.0186 (8)	−0.0062 (8)
Cl2	0.0296 (3)	0.0394 (3)	0.0465 (3)	−0.0015 (2)	0.0254 (2)	−0.0073 (2)
C14	0.0352 (10)	0.0333 (10)	0.0288 (10)	−0.0056 (8)	0.0229 (9)	−0.0045 (8)
C15	0.0320 (10)	0.0318 (10)	0.0222 (9)	−0.0009 (8)	0.0165 (8)	−0.0011 (8)
C16	0.0258 (9)	0.0256 (9)	0.0206 (8)	−0.0045 (7)	0.0137 (7)	−0.0049 (7)
O1	0.0438 (8)	0.0344 (8)	0.0245 (7)	0.0003 (6)	0.0165 (6)	−0.0050 (6)

### Geometric parameters (Å, °)

**Table:**

N1—C1	1.337 (2)	C8—C9	1.381 (3)
N1—C5	1.343 (2)	C8—Cl1	1.7484 (18)
C1—C2	1.383 (3)	C9—C10	1.368 (3)
C1—H1	0.9500	C9—H9	0.9500
C2—C3	1.381 (3)	C10—C11	1.409 (2)
C2—H2	0.9500	C10—H10	0.9500
C3—C4	1.383 (3)	N4—C16	1.368 (2)
C3—H3	0.9500	N4—H4N	0.8698
C4—C5	1.388 (3)	N5—C16	1.338 (2)
C4—H4	0.9500	N5—C12	1.348 (2)
C5—C6	1.526 (2)	C12—C13	1.372 (3)
C6—N4	1.442 (2)	C12—H12	0.9500
C6—N2	1.452 (2)	C13—C14	1.389 (3)
C6—H6	1.0000	C13—Cl2	1.7418 (19)
N2—C11	1.358 (2)	C14—C15	1.365 (3)
N2—H2N	0.8699	C14—H14	0.9500
N3—C7	1.342 (2)	C15—C16	1.413 (2)
N3—C11	1.344 (2)	C15—H15	0.9500
C7—C8	1.375 (3)	O1—H1O	0.8206
C7—H7	0.9500	O1—H2O	0.8206
			
C1—N1—C5	118.11 (16)	C9—C8—Cl1	121.13 (15)
N1—C1—C2	123.50 (18)	C10—C9—C8	118.51 (17)
N1—C1—H1	118.3	C10—C9—H9	120.7
C2—C1—H1	118.3	C8—C9—H9	120.7
C3—C2—C1	118.10 (18)	C9—C10—C11	119.16 (17)
C3—C2—H2	121.0	C9—C10—H10	120.4
C1—C2—H2	121.0	C11—C10—H10	120.4
C2—C3—C4	119.16 (18)	N3—C11—N2	117.63 (15)
C2—C3—H3	120.4	N3—C11—C10	122.19 (16)
C4—C3—H3	120.4	N2—C11—C10	120.16 (16)
C3—C4—C5	119.22 (18)	C16—N4—C6	123.43 (15)
C3—C4—H4	120.4	C16—N4—H4N	117.7
C5—C4—H4	120.4	C6—N4—H4N	118.5
N1—C5—C4	121.90 (16)	C16—N5—C12	117.83 (15)
N1—C5—C6	116.09 (15)	N5—C12—C13	123.00 (18)
C4—C5—C6	121.98 (16)	N5—C12—H12	118.5
N4—C6—N2	110.65 (14)	C13—C12—H12	118.5
N4—C6—C5	110.04 (14)	C12—C13—C14	119.23 (17)
N2—C6—C5	111.98 (15)	C12—C13—Cl2	120.28 (15)
N4—C6—H6	108.0	C14—C13—Cl2	120.48 (14)
N2—C6—H6	108.0	C15—C14—C13	118.88 (17)
C5—C6—H6	108.0	C15—C14—H14	120.6
C11—N2—C6	122.92 (15)	C13—C14—H14	120.6
C11—N2—H2N	117.7	C14—C15—C16	118.96 (18)
C6—N2—H2N	119.3	C14—C15—H15	120.5
C7—N3—C11	117.36 (15)	C16—C15—H15	120.5
N3—C7—C8	123.29 (17)	N5—C16—N4	118.68 (16)
N3—C7—H7	118.4	N5—C16—C15	122.09 (17)
C8—C7—H7	118.4	N4—C16—C15	119.23 (16)
C7—C8—C9	119.47 (17)	H1O—O1—H2O	106.9
C7—C8—Cl1	119.40 (15)		

### Hydrogen-bond geometry (Å, °)

**Table:**

*D*—H···*A*	*D*—H	H···*A*	*D*···*A*	*D*—H···*A*
N2—H2N···N5^i^	0.87	2.15	2.994 (2)	165
N4—H4N···O1^ii^	0.87	2.18	2.993 (2)	156
O1—H1O···N1	0.82	1.98	2.773 (2)	164
O1—H2O···O1^iii^	0.82	1.96	2.758 (2)	163

Symmetry codes: (i) *x*, *y*−1, *z*; (ii) −*x*+1/2, *y*−1/2, −*z*+1/2; (iii) −*x*+1/2, *y*+1/2, −*z*+1/2.
